# Long-term homeostasis and wound healing in an *in vitro* epithelial stem cell niche model

**DOI:** 10.1038/srep43557

**Published:** 2017-02-24

**Authors:** Hideyuki Miyashita, Hiroko Niwano, Satoru Yoshida, Shin Hatou, Emi Inagaki, Kazuo Tsubota, Shigeto Shimmura

**Affiliations:** 1 Department of ophthalmology/Corneal cell biology group, Keio university school of medicine, Tokyo, Japan

## Abstract

Cultures of epithelial cells are limited by the proliferative capacity of primary cells and cell senescence. Herein we show that primary human epithelial cell sheets cultured without dermal equivalents maintained homeostasis *in vitro* for at least 1 year. Transparency of these sheets enabled live observation of pigmented melanocytes and Fluorescent Ubiquitination-based Cell Cycle Indicator (FUCCI) labeled epithelial cells during wound healing. Cell turn over and KRT15 expression pattern stabilized within 3 months, when KRT15 bright clusters often associated with niche-like melanocytes became apparent. EdU labels were retained in a subset of epithelial cells and melanocytes after 6 months chasing, suggesting their slow cell cycling property. FUCCI-labeling demonstrated robust cell migration and proliferation following wounding. Transparency and long-term (1 year) homeostasis of this model will be a powerful tool for the study of wound healing and cell linage tracing.

The mammalian body surface is covered by stratified squamous epithelia, which act as a barrier against the environment. Both continuous cell turnover and wound healing are important for epithelial homeostasis and proper epithelial function. Continuous cell turnover supplies differentiated cells that form a front line against the environment. Wound healing is required to close wounds in order to prevent invasion of microorganisms. Epithelial stem cells have a slow cell cycle and generate transient amplifying cells, which proliferate rapidly and move superiorly to upper layers while undergoing differentiation^1^,^2^. During wound healing, epithelial cells first migrate into the wound, followed by proliferation and stratification^3^.

The corneal epithelium is a stratified squamous epithelium covering the cornea, a transparent tissue of the ocular surface. Unlike the epidermis, ocular surface epithelia, including corneal and conjunctival epithelium, are covered by tear fluid, express transcriptional factor PAX6^4^,^5^, and consist of non-keratinized nucleated cells. Corneal epithelium specifically expresses cytokeratins KRT3 and KRT12 in differentiated cells^6^, while conjunctival epithelium expresses KRT13^7^, which is widely expressed in non-cornified stratified squamous epithelia^8^. Homeostasis of corneal epithelium was proposed by Thoft in 1983 as the X, Y, Z hypothesis; Z is the cell loss from the surface, which is equal to the sum of X (proliferation of corneal epithelial basal cells) and Y (centripetal movement of peripheral cells)^9^. Y in Thoft’s hypothesis is now understood as the proliferation of corneal epithelial stem cells in the limbus, the tissue between the cornea and conjunctiva, which contains blood vessels and melanocytes^10^. Numerous evidences show that the majority of corneal epithelial stem cells are located in the limbus, although several reports suggest the presence of stem cells in the central cornea in addition to the limbus^11^,^12^,^13^,^14^,^15^. Compared to corneal epithelium, the basal cells of the limbal epithelium have a slow cell cycle^16^, show high growth potential^17^,^18^,^19^, do not express differentiation related keratins^6^ and connexins^20^, express specific proteins such as ΔNp63α (truncated isoform of TP63)^21^, KRT15^22^, and others (reviewed in refs 2 and 23).

The human corneal epithelium is unique compared to animal models such as mice. In order to elucidate stem cell/niche interactions in human epithelial cells, an *in vitro* model that mimics the *in vivo* state is required. Organotypic culture (OTC) is the culture of epithelial cells on fibroblast-embedded matrix (dermal equivalent; DEQ) exposed to air in order to enhance stratification (reviewed in ref. 24). OTC shows a well-organized structure, and is often used as an *in vitro* tissue model. Techniques using scaffolds consisting of fibrous, esterified hyaluronic acid filled with fibrin gel and fibroblasts allow an extended culture life for over 15 weeks^25^,^26^. However, DEQ in OTC does not allow cell observation during culture due to its opaque nature. A modified technique reported by Proulx *et al*. consisting of corneal fibroblasts and self-assembled matrix is transparent for at least 2 weeks, however, long-term observation was not performed^27^. On the other hand, epithelial cell sheets used for regenerative medicine are generally cultured without DEQ. Transplanted epithelium can reconstruct damaged ocular surface over a long term, especially when autologous cells sources are used^28^. Although cultured epithelial cell sheets may maintain homeostasis *in vivo* after transplantation, it is difficult to perform detailed examination of these cells due to ethical issues.

In order to obtain an ideal *in vitro* model of the human epithelial stem cell niche, we previously reported that replacing epidermal growth factor (EGF) with fibroblast growth factor 7 (FGF7 or keratinocyte growth factor; KGF) combined with the rho kinase inhibitor Y27632 can extend the culture life of a confluent epithelial cell sheet for up to 3 months (hereafter termed as KY sheet)^29^. Herein, we further show that KY sheets can maintain homeostasis for over 1 year, and can undergo wound healing demonstrated by live fluorescence imaging. The unique transparent property of KY sheets was indispensable for such imaging techniques. In addition, we report changes in cell turnover and the expression pattern of the epithelial stem cell marker during the one-year culture period.

## Results

### Continuous turnover of primary KY sheets for 1 year *in vitro*

**Figure 1:**
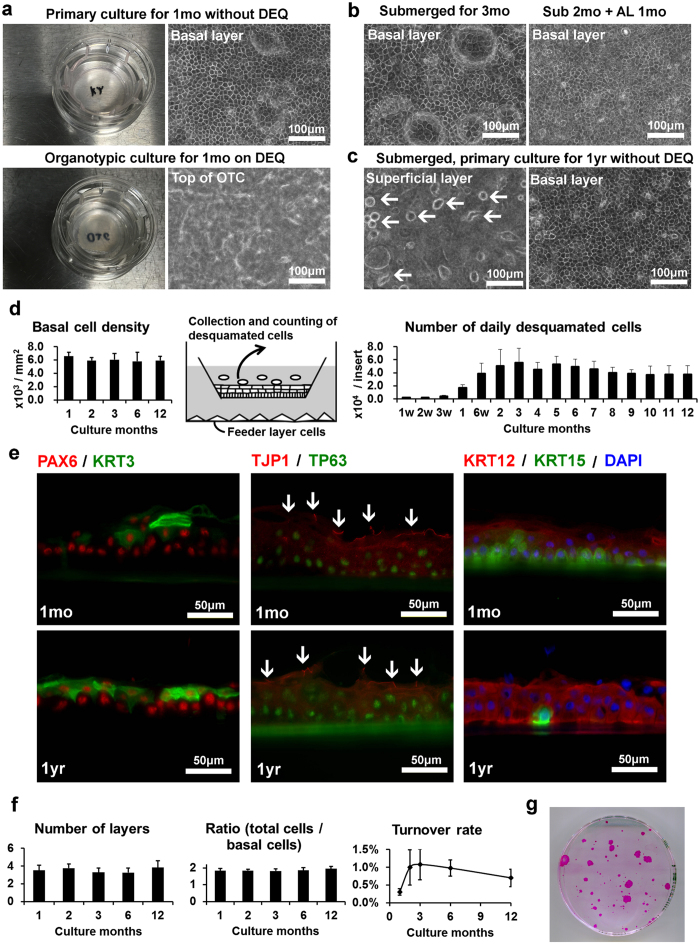
Cultivation of KY sheet for 1 year. (**a**) Epithelial cells from same donor were cultured without DEQ (upper panels) or organotypic cultured with DEQ (lower panels). Digital camera photographs (left panels) and invert microscopy using Objx20 (right panels). (**b**) Influence of air lift culture. KY sheets from same donor were submerged for 3 months or cultured at air/liquid interface for last 1 month. (**c**) Morphology of KY sheet cultured for 1 year as submerged. Arrows indicate desquamated cells floating on the superficial layer. (**d**) Basal cell density (left panel, n = 4–6) and number of desquamated cells (right panel, n = 6–22) at each time point. (**e**) Fluorescent microscopy of primary culture using corneal and limbal epithelium-related markers. Culture periods are indicated in each panel. PAX6, TJP1, and KRT12 are shown in red and KRT3, TP63, and KRT15 are shown in green. Nuclei were counterstained with DAPI shown in blue. White arrows indicate the linear expression of TJP1 between superficial cells. (**f**) Number of cell layers (n = 4–7), ratio of total cell numbers to basal cell numbers (n = 4–8), and turnover rate (n = 6–22) at each time points. (**g**) Colony formation of epithelial cells dissociated from 1 year culture. Colonies in ϕ100 mm indicator dishes were stained with Rhodamin B (red). Error bars indicate S.D.

At first, we set to confirm how long KY sheets could be maintained *in vitro*. We initially set the upper limit of cultivation period at 1 year, and to our surprise, KY sheets were maintained for over 1 year (Fig. 1). Cell morphology in KY sheets was easy to observe by inverted microscopy, whereas cells in OTC were hard to observe except for mesenchymal cells invading into the acellular layer of DEQ during early OTC (Fig. 1a; Supplementary Fig. S1a). Although air-lifting diminished vacuolar structures in suprabasal cells and enhanced differentiation marker KRT3 expression (Fig. 1b; Supplementary Fig. S1b), we cultured KY sheets under submerged conditions in order to simplify the culture method and to reduce variable factors. Basal cell morphology was similar for 1 year (Fig. 1a,b,c; Supplementary Fig. S1c). Even though the culture medium was replaced daily, desquamated cells were observed every day (Fig. 1c, arrows). Feeder layer of human bone marrow derived mesenchymal stem cells also survived throughout the culture period, although cells autonomously embedded into secreted extracellular matrix after 3 months (Supplementary Fig. S1c). Exchanging feeder cells during culture did not seem to influence the condition of KY sheets (Supplementary Fig. S1d).

To confirm whether the epithelium structure changed during culture, basal cell density and the number of desquamated cells were counted at several time points (Fig. 1d, Table 1). Basal cell density was stable throughout the culture period. In contrast, number of daily desquamating cells collected from the supernatant increased from 1 to 3 months, and stabilized thereafter. These results suggest that the total number of epithelial cells was stable throughout the culture period, whereas the flow of cell mass required 2 to 3 months to stabilize. The number of daily desquamated cells was not related to donor age or gender, seeding cell density, tissue storage period (days from donor death to cell preparation) or passage number of feeder cells (Supplementary Fig. S1e).

Since we previously experienced the loss of corneal specific markers during the establishment of a murine cell line^30^,^31^, we confirmed the expression of corneal and limbal markers after 1-year culture (Fig. 1e). Cultures at 1 month were used as control. Immunohistochemistry (IHC) showed that PAX6 was observed in all cells. KRT3 was detected in several suprabasal cells and KRT12 was observed in suprabasal cells and several basal cells. In addition to these corneal lineage markers, tight junction protein 1 (TJP1/ZO1) was observed between superficial cells (Fig. 1e, arrows). HRP permeability assay^32^ showed that KY sheets maintained a functional barrier, as HRP did not intrude into cell sheets without impairing the cell barrier by treatment with 0.02% benzalkonium chloride (BAC)^33^ (Supplementary Fig. S1f). These data suggest that KY sheets maintain cell barrier function. Staining of the epithelial stem cell marker TP63 was observed in almost all cell nuclei. Epithelial stem cell marker KRT15^22^,^34^,^35^,^36^ was expressed uniformly in basal cells at 1 month, but was limited to small cell clusters at 1 year. These results show that expression of limbal markers was stable during the culture, except for KRT15.

Turnover rate signifies the ratio of shedding cells to total cell number. Shedding cells were monitored by counting desquamating cells daily, and total cell number was estimated as the product of (culture area) × (basal cell density) × (the ratio of total cell number to basal cell number). To obtain the ratio of total cell number to basal cell number, we counted cells in cryo-sectioned samples. In addition, we measured the thickness of the cell layer during culture. We found that the number of cell layers and the ratio of total cell number did not change throughout the culture period (Fig. 1f, Table 1). Turnover rate increased from 1 to 3 months (Fig. 1f, Table 1), with the lowest turnover at 1 month compared to 2 to 6 months (p < 0.05, One way ANOVA followed with Sheffe’s F test). Senescence-associated β-galactosidase (SA-β-gal)^37^ was not detected in both early and late cultures (Supplementary Fig. S1g).

To confirm whether KY sheet retained proliferative ability after long-term culture, we performed colony forming assays by using cells dissociated from sheets cultured for 1 year (Fig. 1g). Growing colonies formed at an efficiency of 9.0% ± 3.8% (n = 3), showing that proliferative potential of KY sheets was maintained even after 1-year culture.

### The existence of KRT15-bright cell clusters in KY sheets

**Figure 2:**
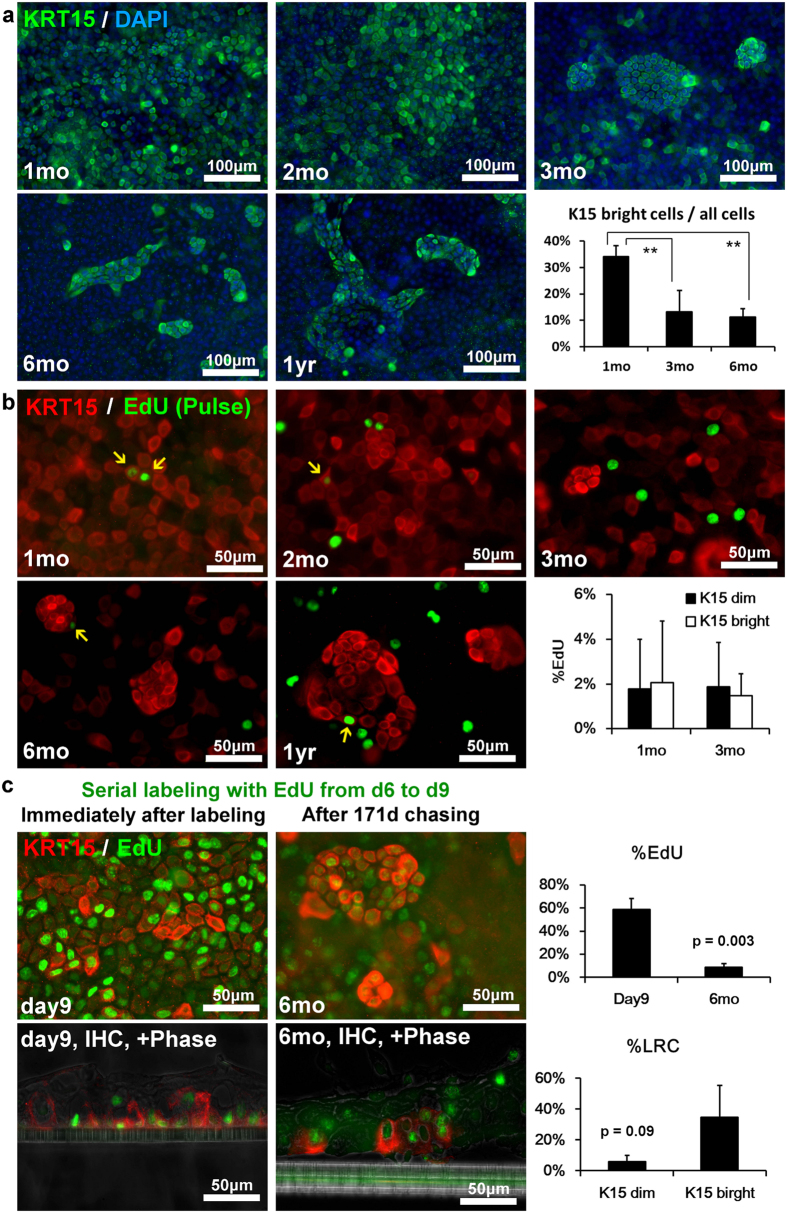
KRT15 expression pattern and pulse labeling, serial labeling, and long term chasing of EdU in KY sheets. (**a**) WMS by using mouse monoclonal anti-KRT15 antibody (green). Nuclei were stained with DAPI. At 3 months, KRT15 bright clusters and KRT15 dim area became apparent. Graph shows rate of KRT15 bright cells (n = 4 at 1 mo and 6 mo, n = 8 at 3 mo). One way ANOVA (p < 0.05) was followed with Sheffe’s F test (**p < 0.01). (**b**) Proliferating cells and KRT15 expression pattern. WMS with KRT15 (red) and EdU (green). EdU was administrated 1 day prior to fixation. Arrows show cells double positive for KRT15 and EdU. Graph shows EdU positive rate in KRT15 dim cells and KRT15 bright cells at 1 month (n = 4) and 3 months (n = 8). (**c**) LRCs and KRT15 expression pattern. WMS and IHC with KRT15 (red) and EdU (green). Semi-confluent cells were administered with EdU for 3 days (d9), and subsequently cultured for additional 171 days without EdU (6 mo). Upper graph showed the EdU positive rate at day 9 and 6 months (n = 4, paired t test), and lower graph shows the LRC rate in KRT15 dim cells and KRT15 bright cells (n = 4, paired t test). All data in graph are represented as mean ± S.D.

Since KRT15 expression was different between 1 month and 1 year, we performed whole mount staining (WMS) to visualize KRT15 expression patterns (Fig. 2a). At 1 month, almost all basal cells expressed KRT15, although expression levels seemed to differ among cells. In several regions, densely packed clusters of KRT15 bright cells were observed. At 2 months, KRT15 expression varied by cell lot; the pattern in several samples resembling that at 1 month, while other lots resembled patterns observed at 3 months. After 3 months, KRT15 dim areas were apparent, which allowed densely packed KRT15-bright cell clusters to be distinguished easily. KRT15-bright cell clusters were observed during the rest of the culture period at 6 months and 1 year. Percentage of KRT15 bright cells was 34.2% ± 4.1% at 1 mo (n = 4), 13.2% ± 8.2% at 3 mo (n = 8), and 11.3% ± 3.0% at 6 mo (n = 4), respectively, and was statistically significant between 1 and 3 months (p < 0.01, One way ANOVA followed with Sheffe’s F test).

To observe the association of cell proliferation with KRT15 expression, several wells were treated with the thymidine analogue EdU 1 day prior to fixation, followed by WMS for EdU and KRT15 (Fig. 2b). Some KRT15 bright cells incorporated EdU (Fig. 2b, arrow), indicating that KRT15 bright cells were not growth-arrested cells. Although EdU uptake seemed to be rare in KRT15 bright cells after 3 months, the number of KRT15 bright cells itself was reduced. EdU staining in KRT15 bright cells and KRT15 dim cells were not significantly different at both 1 month (p = 0.44, n = 4, paired t test) and 3 months (p = 0.49, n = 8, paired t test), respectively.

To investigate the existence of slow cycling cells, we performed label-retaining assay (Fig. 2c). Semi-confluent KY sheets were serially labeled with EdU for 3 days, and chased for 6 months without EdU. Immediately after labeling, 58.8% ± 9.6% cells were positive for EdU, and EdU positive cells significantly decreased to 8.8% ± 3.1% at 6 months (n = 4, p = 0.003, paired t test). KRT15 bright cells appear to contain more LRCs compared to KRT15 dim cells, however the difference was not significant (n = 4, p = 0.09, paired t test). LRCs were spread around KRT15 bright cell clusters, and in some areas a linear progression from KRT15 bright basal cells to KRT15 dim superficial cells was observed (Fig. 2c, lower right panel). This result suggests a cell linage from slow cycling KRT15 bright basal cells to differentiated cells.

### The association of KRT15-bright cell clusters and dendritic melanocytes in KY sheets

**Figure 3:**
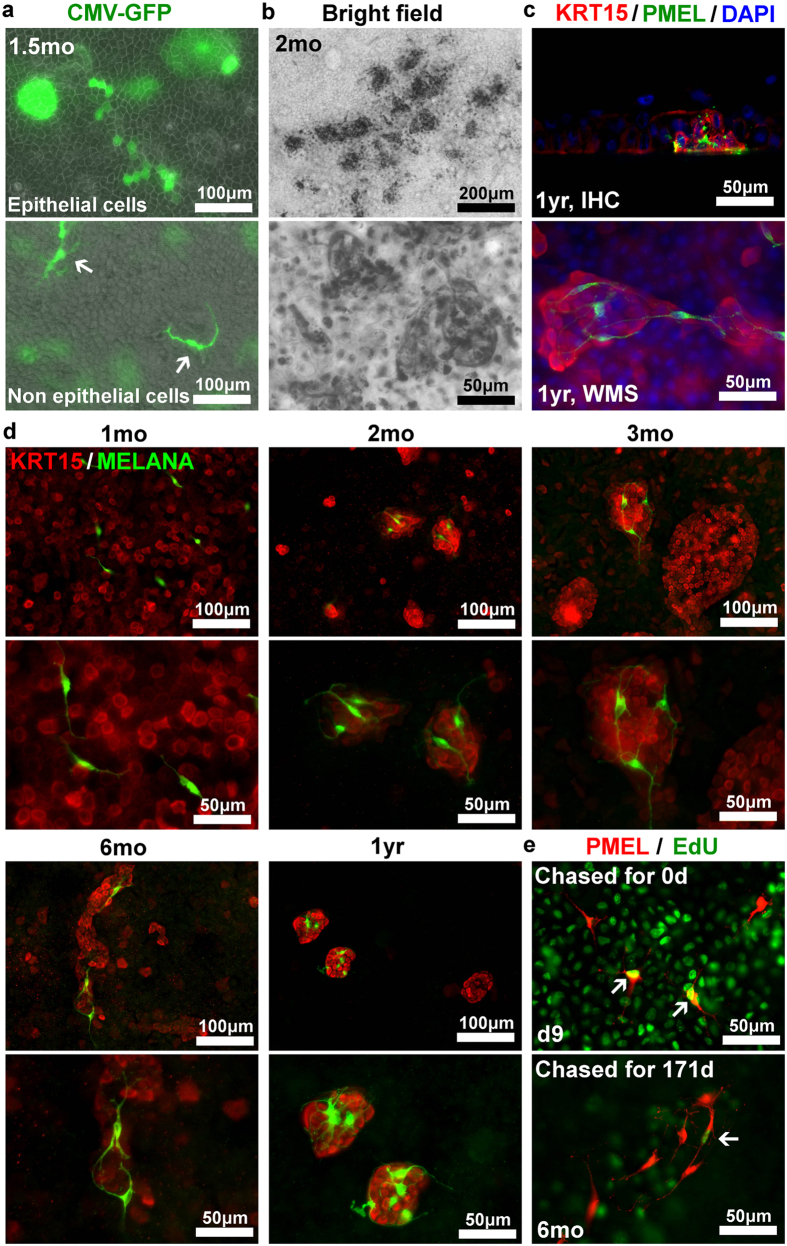
Melanocytes in KY sheets. (**a**) Fluorescent inverted microscopy of primary cultures labeled with lentiviral vector carrying CMV-EGFP. EGFP labeled epithelial cells and EGFP labeled non-epithelial cells (arrow) were shown. (**b**) Spontaneous pigmentation of a primary culture derived from a dense pigmented limbus. Bright field images were taken by invert microscope. Melanocytes and melanin cap of surrounding epithelial cells were pigmented. (**c**) IHC and WMS images of 1 year-cultured primary cells, which was immunostained with KRT15 (red) and melanocyte marker PMEL (green). KRT15 positive cell clusters were surrounded by the dendrite of PMEL positive cells. Nuclei of immunostained samples were counterstained with DAPI (blue). (**d**) WMS images of primary cells cultured for indicated periods. Cells were immunostained with KRT15 (red) and melanocyte marker MELANA (green). Note the presence of KRT15 positive cell cluster without melanocytes. (**e**) PMEL positive melanocytes (red) and EdU positive cells (green) at immediately after serial 3 days labeling (right) and after 6 months chasing (left). Arrow indicates EdU positive melanocytes.

Transparency of KY sheets enabled fluorescent observation of live cells, resulting in the discovery of dendritic non-epithelial cells in CMV-GFP labeled culture (Fig. 3a). Similarly, when we used densely pigmented limbus as a cell source, we observed pigmented epithelial cells associating with pigmented melanocyte-like cells (Fig. 3b). These observations suggest that limbal melanocytes are preserved and functional even after long-term primary culture. One-year cultured KY sheets were immunostained with the melanocyte marker PMEL (Fig. 3c) and MELANA/MART-1 (Fig. 3d). PMEL positive cells were located immediately above the KRT15 bright cell clusters, although limbal melanocytes *in vivo* locate within the basal cell layer. We speculate that this difference is due to the absence of limbal stroma in our culture. Dendrites of MELANA positive cells enwrapped KRT15 bright cell clusters (Fig. 3d). However, KRT15 bright cell clusters without melanocytes were observed, and melanocytes were also located in KRT15 dim areas implying that contact of KRT15 bright cells with melanocytes was not essential for mutual survival, although they tended to associate with each other. In LRC experiments, several melanocytes incorporated EdU after 3 days labeling and retained label after 6 months chasing (Fig. 3e, arrow), suggesting that melanocytes in KY sheets underwent cell cycle slowly.

### Wound healing ability of KY sheets

**Figure 4:**
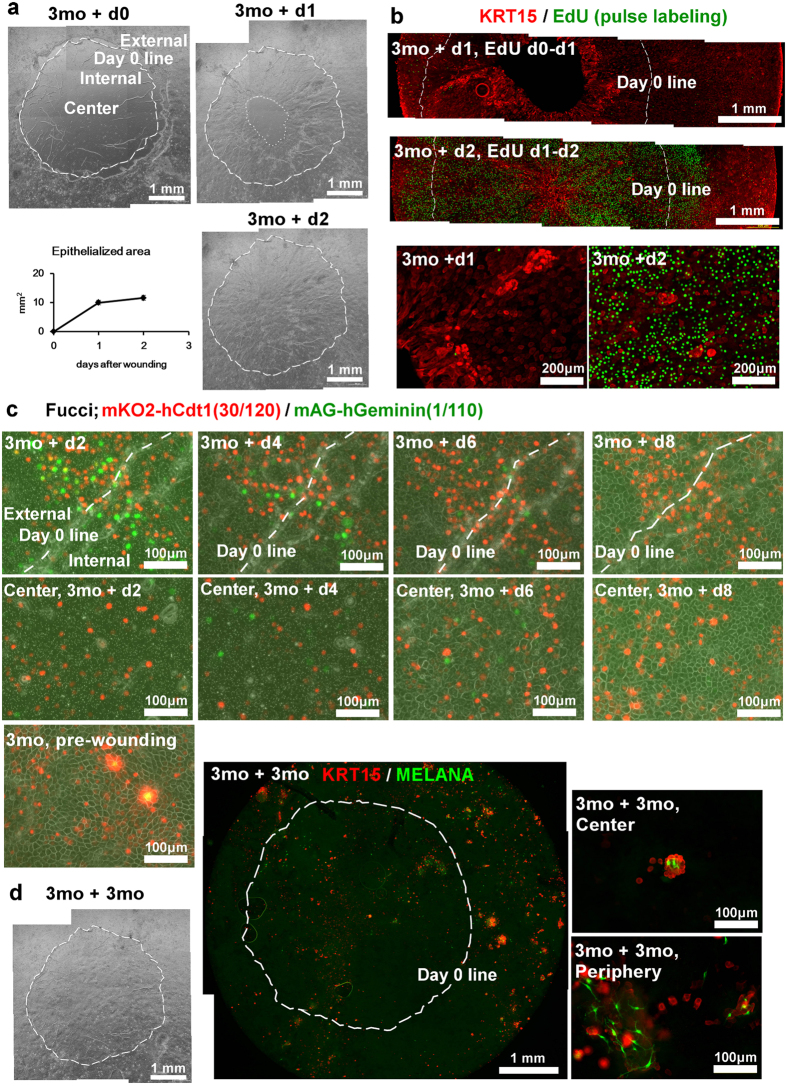
Wound healing ability of KY sheets. (**a**) Stereoscopy micrograph shows the creation of ϕ4 mm wound to KY sheets using forceps. Phase contrast micrograph shows the wounded area of epithelial sheets, cultured for 3 months, created with ϕ4 mm wounds, and cultured for additional indicated periods (+d0 to +d2). Wound shape was marked as a scratch on the plastic surface of the culture insert, and shown as a white dashed line in each panel (day 0 line). White dotted line indicates the edge of the migrating epithelium (**b**,**c**) Proliferation during wound healing. (**b**) Whole mount immunofluorescent microscopy of EdU (green) and KRT15 (red). (**c**) Serial fluorescent micrographs of Fucci transfected primary culture during the wound healing. Epithelial cells on the scratch (border of the original wound, upper panels) and center of the wound (lower panels) are shown. Red mKO2-hCdt1 (30/120) indicates cells in the G_1_ phase and green mAG-hGeminin (1/110) indicates cells in the S, G_2_, and M phase. (**d**) Stereoscopic micrograph and whole mount immunofluorescent micrograph of a 3 months-old wound. KY sheets were stained with KRT15 (red) and MELANA (green).

Since both continuous cell turnover and wound healing are required for epithelial homeostasis, we next examined the wound healing ability of 3-month KY sheets in a steady state. We made circular ϕ4 mm wounds by peeling the epithelium (Supplementary Fig. S2). Epithelial cells migrated into the wound on the next day covering 10.0 ± 0.8 cm^2^ of the wound (Fig. 4a, mean ± S.D., n = 4). Complete epithelization of ϕ4 mm wounds (11.6 ± 0.9 cm^2^) was achieved within 2 days. To monitor cell proliferation during wound healing, wounded cultures were administrated with EdU from wound day 0 to day 1 and from day 1 to day 2, respectively (Fig. 4b). EdU positive cells were sparse at day 1. In contrast, intense labeling of EdU was observed at day 2, expanding from the middle to periphery of the wound. Both KRT15 bright cells and KRT15 dim cells migrated into epithelized area. Although EdU was mostly located in KRT15 dim cells, several KRT15 bright clusters were also positive for EdU. To observe proliferation for longer periods, KY sheets were labeled with lentiviral vectors carrying Fucci at culture day 1, and wounded after 3 months culture, followed with serial observation (Fig. 4c). In Fucci cells, green fluorescence of mAG-hGeminin (1/110) indicates cells in the S, G_2_, and M phases, whereas red fluorescence of mKO2-hCdt1 (30/120) indicates cells in G_1_ phase^38^. Before wounding, Fucci red cells were predominantly observed, whereas green fluorescence was rarely observed. After wounding, Fucci green cells were observed from day 2 after wounding, but decreased at day 4 in the periphery as well as in the center of the wound by day 8. Fucci red cells were observed throughout the wound healing process. These results show that transient proliferation occurred for several days during wound healing, and also demonstrate that the transparency of KY sheets allows fluorescence observation in living cells.

To confirm whether a normal epithelium including putative stem cell compartments regenerated following wounding, cells were cultured for an additional 3 months (Fig. 4d). An intact epithelium was observed covering the originally wounded area, and KRT15 bright cells associating with melanocytes were also observed, suggesting that regenerated epithelium maintained a stem cell compartment.

## Discussion

We successfully demonstrated how primary culture of human limbal epithelial cell sheets reached a steady state within 3 months, and showed continuous cell turnover and wound healing ability for at least 1 year. These results indicate the homeostasis of cultured epithelium *in vitro*, and strongly suggest the maintenance of stem cells in niche-like structures. Long-term culture of epithelial cells has been known for over two decades, with serial passage of epidermal keratinocytes for over 200 days (17–18 passages, 140 cell population doublings)^39^ and for 2–3 months (14 passages, 80–100 cell population doublings) in limbal epithelial cells^19^. In these serial passage experiments, cells are subcultured before they reach confluence, and confluent cells lost viability and decreased in number during prolonged culture^40^. In contrast, KY sheets maintained total cell number for 1 year. As shown in our previous report, this difference was due to replacing EGF with KGF and Y27632^29^. Administration of EGF from the seeding medium decreases CFE^39^. EGF stimulates proliferation, but also stimulates cell motility and diminishes KRT3 expression in limbal epithelial cells^41^. In our previous study, cells cultured with EGF from initial seeding did not survive over 3 months^29^. KGF/FGF7 stimulates the proliferation of keratinocytes^42^ and limbal epithelial cells without increasing cell motility^41^, and enhances the expression of p63 via the p38 pathway in limbal epithelial cells^43^. EGF seems to have a superior effect on epithelial cells compared to KGF, since EGF stimulates epithelial cell migration even in the presence of KGF^41^. Y27632 is known to inhibit keratinocyte differentiation in cell suspension^44^, immortalize keratinocytes^45^, and increase CFE of limbal epithelial cells^29^,^46^. Y27632 cannot preserve KRT15 expression in EGF-treated cultures, while Y27632 increased CFE in KGF supplemented-cultures^29^. From these observations, removing EGF while supplementing KGF and Y27632 may be crucial for the maintenance of primary cultures over long term.

Recently, several methods were developed to induce corneal epithelial linage cells from human embryonic stem cells (ESCs)/induced pluripotent stem cells (iPSCs)^47^,^48^,^49^,^50^,^51^,^52^,^53^,^54^,^55^. However, since ESC and iPSC are pluripotent, other cell types may be induced together with corneal epithelial cells. Purification and detailed characterization is required to prove that the induced cells are truly of corneal epithelial linage^51^. One of the greatest advantages of our KY sheets is the simplicity of the technique. Compared to ESC/iPSC cultures, our method only requires few steps to isolate cells from the limbus and co-culture with feeder cells under submerged conditions. The limbal origin of cells also ensures that the cells are of corneal epithelial lineage.

Transparency of KY sheets is useful for cell linage analysis and investigating cell signaling in specific cells, when combined with proper fluorescent reporters. Despite its simplicity, LRC rate was 8.8% after 6 months chasing in KY sheet, compared to 0.9% after 6 weeks chasing in OTC^26^, suggesting that cells in KY sheets had a slower cell cycle compared to OTC. In addition, KY sheets contained colony-forming cells even after 1 year culture, proving that KY sheets maintained stem/progenitor cells *in vitro* throughout culture. Storage period of donor tissue did not influence the quality of KY sheet states, which may allow flexibility of experiment schedules. Our culture protocol can also be used for other experiments requiring human corneal epithelial cells. For example, culture inserts can be treated with different substrates to study the effects of biomechanical stress, or stiffness of extracellular matrix on cell homeostasis^56^.

Compartmentalization of stem cells following confluency was reported in organotypic culture of primary human epidermal keratinocytes^57^. Similarly, we found densely packed cell clusters expressing epithelial stem cell marker KRT15^34^,^35^,^36^ in KY sheets. KRT15 negative basal cells were positive for KRT12, showing characteristic features of corneal epithelium, which is differentiated from limbal epithelium. Thus the steady state KY sheet may be used as an ocular surface model including both corneal epithelium and limbal epithelium. The mechanism by which KRT15 was maintained in densely packed clusters during culture is unclear. One feasible explanation is that KRT15 bright clusters were protected by the niche, while KRT15 bright single cells were not, thus driving KRT15 bright single cells to differentiate and become lost during culture. There is also the possibility that KRT15 bright clusters were formed from cell aggregates at the time of seeding. Further studies using fluorescent probes driven by human KRT15 promoters^58^ may elucidate the mechanism cluster formation.

Wound healing process of corneal epithelium consists of an initial phase and a closure phase (Reviewed in ref. 3). The closure phase starts with the cell migration without mitosis, followed by the cell proliferation and differentiation. Similarly, KY sheets showed the cell migration without mitosis at day 1 after wounding, followed by proliferation at day 2 after wounding, indicating the usefulness of KY sheets as wound healing model. Interaction between epithelial cells and mesenchymal cells also occurs during the wound healing (Reviewed in refs 3,59). Since KY cultures contained a feeder layer consisting of mesenchymal cells, crosstalk between epithelial cells and mesenchymal cells via soluble factors may have occurred during the wound healing assay. A limitation of our model is that unlike stem cells *in vivo* that are presumably anchored to the stromal niche, our cells *in vitro* are not in contact with the feeder cells. This may explain the migration of KRT15 bright clusters during wound healing.

Melanocytes are a niche candidate to support limbal epithelial stem cells^10^,^60^,^61^, and dendritic melanocytes surrounding several KRT15 bright cell clusters were observed in KY sheet. However, KRT15 bright clusters not associated with melanocytes were also observed, suggesting that other niche factors may maintain KRT15 bright clusters. Since melanocytes were a serendipitous observation, conditions that allow these cells to be maintained in culture need to be elucidated. It is possible that the re-association of epithelial stem/progenitor cells and melanocytes may have occurred immediately following dissociation. While not within the scope of this study, the transparent nature of KY sheets should allow the study of interaction between human epithelial cell and melanocytes *in vitro*.

In conclusion, we showed the homeostasis of primary cultured human limbal epithelial cells for 1 year *in vivo* without the use of dermal equivalent, maintained by continuous cell turnover with the maintenance of KRT15 bright cell clusters often associating with melanocytes and wound healing ability. This protocol will be a powerful tool in the study of stem cells, wound healing and epithelial/melanocyte interaction, and cell linage analysis using fluorescent reporters. While our protocol was not intended for clinical use, long-term maintenance of cells sheets with a proven stem cell population may also open doors to a new generation of regenerative medicine techniques.

## Methods

### Primary culture of human limbal epithelial cells with KGF and Y27632

We cultured human limbal epithelial cells as previously reported^29^. In brief, primary culture of human limbal epithelial cells were separately co-cultured with feeder layer cells by using cell culture inserts^62^ with medium containing KGF and Y27632^29^. We used human mesenchymal stem cells (marrow adherent stem cells, MASCs, a kind gift from Dr. McGrogan, San Bio Inc., Mountain View, CA) as feeder layer cells^63^. MASCs were established as adherent cells from bone marrow, and characterized as CD29 (+), CD90 (+), CD105 (+), CD31 (−), CD34 (−), and CD45 (−) cells by the provider. MASCs at passage 5.4 ± 1.5 (mean ± S.D.) were cultured in 6 well plates, and fed with MEM-α containing 10% fetal bovine serum (FBS) every 3–4 days. Confluent MASCs were treated with Mitomycin C (4 μg/mL) for 2 hours at 37 °C and subsequently used for epithelial culture.

Human limbal epithelial cells were isolated from U.S. eyebank eyes (Sightlife, Seattle, WA). Donor age was ranged from 20 to 75, 60.6 ± 10.8 years old (n = 71, male = 39, female = 32). Eyes were shipped in cold preservation medium (Optisol, Bausch & Lomb, Rochester, NY) in a cornea viewing chamber (Bausch & Lomb), and stored at 4 °C. Mean time from death to cell isolation was 11.9 ± 3.6 days. After the removal of excess tissue, limbal epithelium was separated from stroma by Dispase II treatment (final 4U/mL, Roche, Basel, Switzerland) at 37 °C for 1 hour, followed with the dissociation by pipetting to obtain the mixture of single cells and cell aggregates. In 3 experiments, epithelium was further dissociated by enzyme treatments (TrypLE Express, Gibco) for 30 min at 37 °C, followed with passing through 40 μm nylon mesh (Cell strainer, Falcon, Corning Incorporated, Corning, NY) to remove large cell aggregates. Dissociated cells were seeded in plastic cell culture inserts (3450, Corning), subsequently co-cultured with feeder cells in the bottom of paired well. Since harvested cell number varied among donors (2.8 ± 1.8 × 10^5^), isolated cells were evenly separated to 3 inserts. Average seeding cell density was 9.1 ± 7.3 × 10^4^ cells/insert.

Components of epithelial medium were as follows; 96% DMEM/F12, FBS (4%), recombinant human FGF7 (KGF, 10 ng/mL), Y27632 (10 μM), insulin (10 μg/mL), hydrocortisone (0.5 μg/mL), tri-iodo-thyronine (2 nM), isoproterenol (250 ng/mL), and antibiotics. Medium was changed at day 3, day 5, and every day after day 7.

### Organotypic culture

DEQ was constructed by using MASCs and a commercially available collagen type I kit (acidic collagen type I from porcine tendon, KP-7000, Nitta-gelatin Inc., Tokyo, Japan). At first, 1 mL of acellular collagen layer was reconstructed on cell culture insert. After gelation, 3 mL of cellular layer was cast on the acellular layer^64^. Each DEQ contained 5 × 10^5^ of MASCs and 0.7 mg/mL of collagen type I^65^. DEQs were fed with MEM-α containing 10% FBS at day 3 and day 6. Human limbal epithelial cells were cultured in the 25 cm^2^ flask with feeder layer of MMC treated NIH/3T3, and semi-confluent epithelial cells (P0-P1) were passaged on 1wk-old DEQ at a density of 5 × 10^5^ epithelial cells/DEQ. OTCs were cultured as submerged for 4 days^65^, followed with air lift culture for additional 1 month.

### Transfection of lentiviral vectors

Lentivirus packaging vectors pCAG-HIVgp and pCMV-VSV-R-RSV-Rev and lentiviral vector expressing GFP by CMV promoter (pCS-CDF-CG-PRE) were provided by Dr. Hiroyuki Miyoshi (Keio University School of Medicine, Dept. of physiology) via RIKEN Bio Resource Center (Tsukuba, Japan). Fucci vectors, mAG-hGeminin (1/110)/pCSII-EF-MCS and mKO2-hCdt1 (30/120)/pCSII-EF-MCS were provided by Dr. Atsushi Miyazaki and Dr. Asako Sakaue-Sawano (RIKEN Brain Science Institute, Saitama, Japan). Passaged 293 T was lipofected with plasmids at day1, after which medium was exchanged with epithelial medium at day 2, and supernatant was harvested as lentiviral vector solution at day 4. Primary cells were transfected at culture day 1 for Fucci or at semi-confluence for CMV-GFP. Fluorescence was observed using an inverted fluorescent microscope.

### EdU pulse labeling, serial labeling and the chasing of LRCs

For the pulse labeling of proliferating cells, EdU (10 μM, Thermo Fisher Scientific, Waltham, MA) were administrated to the culture 24 hours before fixation. For LRC assay, EdU (1 μM) were administrated to semi-confluent cells (day 6) for serial 3 days. One of triplicated inserts was fixed immediately after serial labeling, and remaining inserts were subsequently cultured for an additional 171 days without EdU. EdU was detected by Alexa flour-488 conjugated azide (Click-iT kit, Thermo Fisher), followed by immunostaining.

### Immunostaining and EdU detection

For IHC, cultures were cut with a ϕ6 mm trephine blade (Kai Industries Co., Ltd., Gifu, Japan). Ten μm thick cryosections were fixed with ice-cold 4% paraformaldehyde (PFA) in PBS for 5 min. Sections were treated with PBS containing 0.1% Triton-X and 10% normal donkey serum to block non-specific staining. For WMS, cultures were fixed with ice-cold 4% PFA for 10 min, cut by ϕ6 mm trephine, and permeabilized with 0.5% Triton-X in PBS at RT for 20 min. Mouse monoclonal antibodies for KRT3 (sc-80000, AE5, Santa Cruz Biotechnology, Inc., Santa Cruz, Dallas, TX), KRT15 (ab-1385, LHK15, Abcam, Cambridge, MA), p63 (sc-8431, 4A4, Santa Cruz), PMEL (sc-52704, NK1/betab, Santa Cruz), chicken anti-KRT15 antibody (PCK-153P, Covance, Princeton, NJ), and rabbit antibodies for KRT12 (sc-25772, Santa Cruz), PAX6 (RPB-278P, Covance), MELANA (MAB1656, Abnova, Taipei, Taiwan), TJP1(402200, Thermo Fisher) were used as primary antibodies. Mouse IgG1 (MOPC21, M5284, Sigma-Aldrich) and mouse IgG2a (UPC-10, M5409, Sigma-Aldrich) were used as isotype control. Alexa fluor 488 and 555 conjugated antibodies (Thermo Fisher) were used as secondary antibodies. IHC sections were treated with antibodies for 1 hour, whereas WMS samples were treated for 2 hours. Nuclei were counterstained with DAPI. Images were obtained by fluorescent microscope.

### Measurement of layer numbers, ratio of total cell number to basal cell number, and basal cell density

DAPI images of IHC and WMS under objective power x20 were used for cell counting. The number of cell nuclei was counted by using cell counter in Image J 1.45 s software (NIH). Four to seven different lots were used in each time point. Number of layers was counted as the nuclei number in the vertical direction from basal cells to superficial cells in IHC images. Ratio of total cell number to basal cell number was calculated by using total nuclei number in basal layer and total nuclei number in all suprabasal layers in each IHC image; total cell number was calculated as the sum of basal cell number and suprabasal cell number. Basal cell density was calculated by the number of nuclei in each WMS image field (1.48 × 10^−1^ mm^2^ at Objx20).

### Calculation of daily desquamated cell number and turnover rate

Supernatants from each cell culture insert were collected by P1000 micropipette. Volume of supernatant was also measured by P1000. Number of desquamated cells was counted by hemocytometer at day 7 (n = 4), day 14 (n = 4), day 21 (n = 6), day 27–29 (n = 19), day 41–43 (n = 16), day 59–61 (n = 21), and every 30 days to day 359–361 (n = 6–22). The average number of desquamating cells during 3 continuous days was recorded as the data for each time point, except for 1–3 weeks. Turnover rate was estimated as (the number of daily desquamated cells)/(the mean total cell number in each insert). Total cell number in each insert was calculated as (basal cell density) × (the ratio of total cells to basal cells) × (the area of cell culture insert, 4.67 cm^2^).

### Colony forming assay

Primary cultures at 1 year were sampled for IHC and WMS without fixation, and remaining cells were treated with cell dissociation buffer (TrypLE express) with Y-27632 at 37 °C for 30 min. After passing through a 40 μm cell strainer to remove cell aggregates, cells were seeded on NIH/3T3 feeder-prepared 100 mm dishes at a density of 1 × 10^3^ cells per dish. Dishes were cultured until colonies became apparent (18 days), and fixed with buffered 10% formalin and stained with Rhodamin B to visualize colonies. Colony formation efficiency was calculated as the percentage of colonies per seeded cell number. Four indicator dishes were used for each lot, and three independent experiments were performed.

### Wound healing assay

KY sheets cultured for at least 3 months were used for wound healing assays. Culture plastic dishes were marked with ϕ4 mm trephines, and cell culture inserts with KY sheets were moved onto a dish (Supplementary Fig. 2). A section of the cell sheet above the mark was peeled off with fine forceps (No. DU-5, Dumont, Montignez, Switzerland) under a stereomicroscope. The shape of the wound was marked on the plastic insert by scratching with forceps. One to four wounds were created in each insert. After washing with culture medium twice, wounded epithelia were returned to the feeder prepared wells and co-cultured. Phase contrast images were merged by raster graphics editor (GIMP 2, The GIMP Development Team). To measure the epithelialized area, day 0 line was scratched on the underside of the cell culture insert, and day 1 line observed as the border of epithelial cells were traced by raster graphics editor and the area between the lines was measured by ImageJ.

### Statistical analysis

Two groups were analyzed by paired t test. Groups over 3 were analyzed by one way ANOVA followed with Sheffe’s F test, performed by excel software and add-in Statcell. Statistical significance was set as p < 0.05.

Methods for HRP permeability assay and SA-*β*-galactosidase staining are provided in Supplementary Information.

## Additional Information

**How to cite this article:** Miyashita, H. *et al*. Long-term homeostasis and wound healing in an *in vitro* epithelial stem cell niche model. *Sci. Rep.* **7**, 43557; doi: 10.1038/srep43557 (2017).

**Publisher's note:** Springer Nature remains neutral with regard to jurisdictional claims in published maps and institutional affiliations.

## Supplementary Material

Supplementary Information

## Figures and Tables

**Table 1:** Numeric value.

Culture periods	1 mo	2 mo	3 mo	6 mo	12 mo
Desquamated cells/insert/day	1.7 ± 0.5 × 10^4^ (n = 19)	5.1 ± 2.5 × 10^4^ (n = 21)	5.6 ± 2.2 × 10^4^ (n = 22)	4.9 ± 1.2 × 10^4^ (n = 11)	3.8 ± 1.3 × 10^4^ (n = 6)
Basal cell density (cells/mm^2^)	6.6 ± 0.6 × 10^3^ (n = 6)	5.9 ± 0.5 × 10^3^ (n = 4)	6.0 ± 0.9 × 10^3^ (n = 11)	5.8 ± 1.4 × 10^3^ (n = 8)	5.9 ± 0.7 × 10^3^ (n = 6)
Number of layers	3.5 ± 0.6 (n = 4)	3.8 ± 0.5 (n = 4)	3.3 ± 0.5 (n = 7)	3.3 ± 0.5 (n = 4)	3.8 ± 0.8 (n = 6)
Ratio of basal cells to total cells	1.8 ± 0.1 (n = 4)	1.8 ± 0.1 (n = 4)	1.8 ± 0.1 (n = 5)	1.9 ± 0.2 (n = 5)	2.0 ± 0.1 (n = 6)
Daily turnover rate	0.30% ± 0.09% (n = 19)	0.99% ± 0.49% (n = 21)	1.08% ± 0.43% (n = 22)	0.98% ± 0.23% (n = 11)	0.70% ± 0.25% (n = 6)

Numeric values collected at indicated periods. Mean ± S.D.
