# Supplementary material for: Long-term homeostasis and wound healing in an in vitro epithelial stem cell niche model
Source: Sci Rep. 2017 Feb 24;7:43557. doi: 10.1038/srep43557 (PMC5324070; doi:10.1038/srep43557)
Supplement: Supplementary Information [file srep43557-s1.pdf]

Title;

Long-term homeostasis and wound healing in an *in vitro* epithelial stem cell niche model

Names of authors;

Hideyuki Miyashita, Hiroko Niwano, Satoru Yoshida, Shin Hatou, Emi Inagaki, Kazuo Tsubota, and Shigeto Shimmura*.

Names of institution;

Department of ophthalmology/Corneal cell biology group, Keio university school of medicine, Tokyo, Japan

*Corresponding Information;

shige@z8.keio.jp

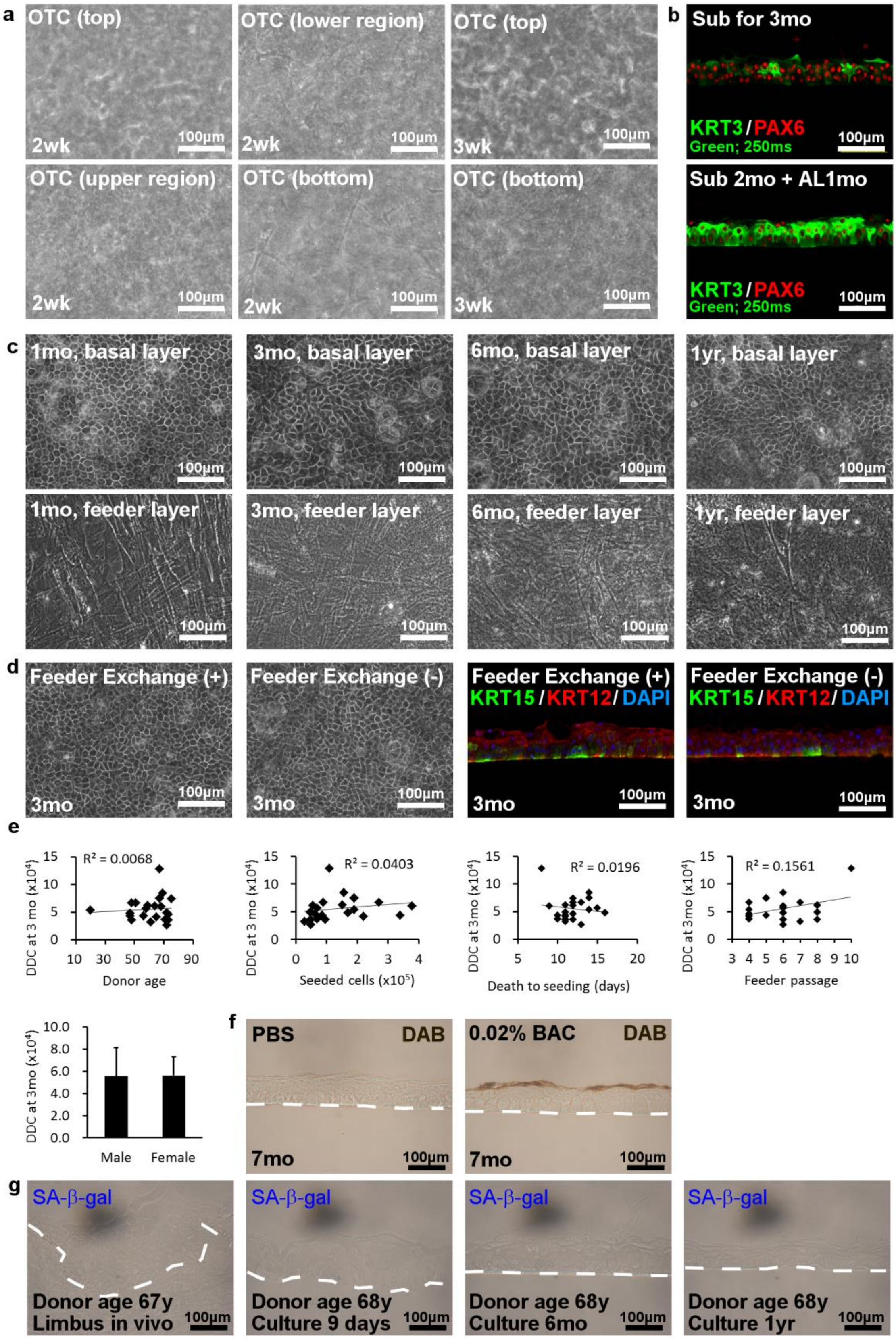
a
OTC (top)
2wk
100μm
OTC (lower region)
2wk
100μm
OTC (top)
3wk
100μm
OTC (upper region)
2wk
100μm
OTC (bottom)
2wk
100μm
OTC (bottom)
3wk
100μm
b
Sub for 3mo
KRT3/PAX6
Green; 250ms
100μm
Sub 2mo + AL1mo
KRT3/PAX6
Green; 250ms
100μm
c
1mo, basal layer
3mo, basal layer
6mo, basal layer
1yr, basal layer
1mo, feeder layer
3mo, feeder layer
6mo, feeder layer
1yr, feeder layer
100μm
d
Feeder Exchange (+)
Feeder Exchange (-)
Feeder Exchange (+)
KRT15/KRT12/DAPI
Feeder Exchange (-)
KRT15/KRT12/DAPI
3mo
100μm
e
DDC at 3 mo (x10⁴)
R² = 0.0068
Donor age
R² = 0.0403
Seeded cells (x10⁵)
R² = 0.0196
Death to seeding (days)
R² = 0.1561
Feeder passage
DDC at 3mo (x10⁴)
Male
Female
f
PBS
DAB
7mo
0.02% BAC
DAB
7mo
100μm
g
SA-β-gal
Donor age 67y
Limbus in vivo
SA-β-gal
Donor age 68y
Culture 9 days
SA-β-gal
Donor age 68y
Culture 6mo
SA-β-gal
Donor age 68y
Culture 1yr
100μm

Supplementary Fig. S1

(a) Microscope image of OTC, consisting of epithelial layer (top), collagen layer containing MSCs (upper region), and acellular collagen layer (lower region and bottom). (b) IHC of KRT3 (green) and PAX6 (red) in KY sheets submerged for 3 months or submerged for 2 months followed by air-lifting for 1 month. (c) Cell morphology of basal layer and feeder layer during 1-year culture. (d) Microscope image and IHC of KRT15 (green) and KRT12 (red) in KY sheets with/without exchanging to new feeder cells. Nuclei were counterstained with DAPI (blue). (e) Scatter plots of the number of daily desquamated cells (DDC) at 3 months and seeded cell number, donor age, days from donor death to cell seeding, feeder passages, and donor gender. n=22. (f) HRP permeability assay. BAC was used to impair cell barrier. White dashed lines shows the basal membrane of epithelial cells. PBS or 0.02% BAC was applied in the upper well for 10 min, followed by the addition of HRP in the upper well for 45 min. HRP was visualized by DAB. HRP was observed in the superficial cells of BAC treated KY sheets, whereas PBS treated KY sheets did not show HRP infiltration. (g) SA-β-gal staining of human limbus (donor age 67y) and KY sheets from same donor (donor age 68y) cultured for 9 days, 6 months, and 1 year. Blue staining was not detected in any of the specimens.

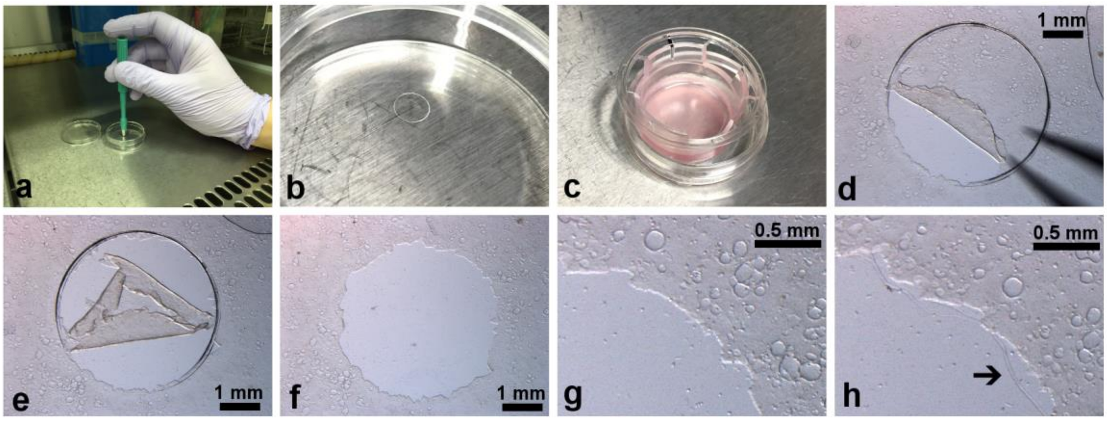

Supplementary Fig. S2

Wounding procedure. Four mm diameter circular marks were created on the 35 mm dish using a trephine (a, b). Culture inserts were placed on the dish (c), followed by peeling of the epithelium using fine forceps along the 4 mm mark (d-f). To record the wound shape, inserts were scratched along the border of the wound (g, h). Arrow indicates the scratch mark.

Supplementary methods

HRP permeability assay

HRP permeability assay was performed to 7-months cultured KY sheets according to the previous report with slight modifications [1]. After the removal of medium from upper wells, 1 mL of PBS or BAC (final 0.02% in PBS, B6295, Sigma) were loaded in the upper wells, and incubated for 10 min at 37˚C [2]. After washing with PBS three times, 100 µL of HRP (final 100 µg/mL in PBS) was loaded in the upper wells, and incubated for 45 min in CO_2 incubator at 37˚C. Subsequently samples were fixed with 2.5% glutaraldehyde in PBS at 4˚C overnight, and washed three times with PBS. After the treatment with 0.2% hydrogen peroxide for 30 min at RT, samples were treated with DAB substrate kit (SK-4100, Vector laboratories, Burlingame, CA) to visualize HRP. Cryosections of DAB stained samples were observed by inverted microscope with digital camera.

SA-β-galactosidase staining

KY sheets from 3 donors (age 68, 69, and 47) were cultured for 9 days and 6 months. Ten micrometer cryosections were fixed with 1% formalin in PBS for 1 min, and subsequently stained with commercially available kit (Senescence detection kit, K320-250, BioVision Inc., Milpitas, CA) overnight at 37 ˚C. Color images were taken by inverted microscope with digital camera

References for supplementary methods

1 Ban, Y. *et al*. Comparison of ultrastructure, tight junction-related protein expression and barrier function of human corneal epithelial cells cultivated on amniotic membrane with and without air-lifting. *Exp Eye Res* **76**, 735-743 (2003).

2 Nakagawa, S. *et al.* Toxicity evaluation of antiglaucoma drugs using stratified human cultivated corneal epithelial sheets. *Invest Ophthalmol Vis Sci* **53**, 5154-5160, doi:10.1167/iovs.12-9685 (2012).
